# Assessment of Elements in Black Tea Infusions by Brewing Method in Terms of their Nutritional Value

**DOI:** 10.1007/s12011-025-04629-7

**Published:** 2025-04-28

**Authors:** Maria Długaszek, Jadwiga Mierczyk, Wojciech Skrzeczanowski, Maria Długaszek

**Affiliations:** Institute of Optoelectronics, Military University of Technology, Sylwestra Kaliskiego 2, 00 - 908 Warsaw 46, Warsaw, Poland

**Keywords:** Black tea infusions, Brewing time, PH, Elements, Atomic absorption spectrometry, Nutritional value

## Abstract

Tea infusions contain many valuable bioactive compounds and elements. The research is aimed at examining them in terms of nutritional value and determining the optimal conditions for their preparation. The study determined the content of Ca, Cu, Fe, K, Mg, Mn, Na, Zn, and Al in infusions of black tea, including bagged and loose leaf. An atomic absorption spectrometer was used for quantitative instrumental analysis. The obtained results were subjected to statistical analysis including PCA (Principal Components Analysis). The brewing time was 5, 10 and 20 min, and the pH value of the infusions was 2 and 6. It was found that, generally, both parameters increased the content of elements in infusions. The amount of elements extracted from 1 g of tea was within the range: Ca- ND (not detected)-7.23 mg, K- 15.1–32.3 mg, Mg- 0.11–0.86 mg, Na- ND-2.85 mg, and Al- ND- 1028 µg, Cu- 1.24–11.02 µg, Fe- ND-36.13 µg, Mn- 97.3–541.6, and Zn- 6.18–22.43 µg. These data were compared with the results for green tea infusions presented in a previous study. The results of this study suggest that the amount of Al and Mn in tea infusions should be controlled.

## Introduction

Tea leaves (*camellia sinensis*) are a source of many valuable nutrients. These include, among others, proteins, carbohydrates, chlorophyll, carotenoids, pectins, fiber, organic acids, methylxanthines (caffeine, theobromine, theanine, etc.), vitamins, minerals and polyphenols.

Tea infusions containing these compounds have many health-promoting properties, such as anti-inflammatory and anti-cancer. They can reduce the risk of many disturbances, including cardiovascular disease, and are helpful in treating, among others, respiratory, skin, liver, and arthritis diseases. It has been shown that they can also prevent the development of type 1 diabetes and improve the immune system [1, 2].

The content of the compounds depends, to a large extent on technological procedures used across all processing stages, including the production of the end food product [1, 2]. In short, the production of green tea includes the withering, rolling and drying of leaves, while black tea includes withering, rolling, oxidation of polyphenols ("fermentation") and drying. Polyphenols present in green tea leaves are mainly flavonoids, i.e., flavan- 3-ols (catechins, gallocatechins), flavonols and their glycosides (including quercetin, rutin, kaempferol). As a result of enzymatic oxidation of simple polyphenols, mainly catechins, they are transformed into larger complexes: theaflavins, thearubigins and others which are present in black tea leaves [1, 2]. The chemical composition of green and black tea leaves does not differ much in terms of dry and organic matter, crude protein and ash content [3]. Significant differences between teas were observed in the total content of polyphenolic compounds and tannins (higher amounts in green tea leaves) and flavonoids (higher in black tea) [3–5]. Polyphenolic compounds have antioxidant properties, and it is mainly thanks to them that tea infusions have anti-cancer and health-promoting effects [2]. It was shown that green tea leaves have a higher antioxidant capacity than black teas [5], and in their infusions, the authors [6] identified similar or higher antioxidant activity in green tea infusions [4, 7]. Dobrinas et al. [8] suggested that the total phenolic content of tea beverages is correlated with antioxidant activity.

During the preparation of tea infusions, many compounds as well elements are leached from the leaves into the beverage. The degree of elements extraction from leaves into the infusion is mainly determined by their quantity in the raw material and the solubility of their compounds, along with brewing time, water temperature, the tea-to-water ratio and the pH value of the infusion [3, 4, 8, 9].

Tea leaves contain large amounts of elements such as K, Na, Ca, Mg, Al and Mn. Elements in tea infusions occur in combination with both organic and inorganic matter. They can be present in the cationic form and non-cationic fractions. Certain elements like Mg, Mn, and Rb are present mainly in cationic form in infusions, but also others as Ca, Fe, Co, Ni, Cu, Sr, and Ba. Some of them may be present in the non-cationic fraction. However, Fe, Ni, Cu and Zn in non-cationic form may occur in combinations with organic compounds. Moreover, the authors stated that aluminum was located in connections with compounds of 4000–6000 Da and 6500–8500 Da in molecular weight. Moreover, it is probable that the element occurs mainly in complexes with thearubigin [10]. Polyphenols are the main organic ligands that bind metallic elements through hydroxyl, carboxyl and phenolic groups [11]. Among the inorganic anions, the most leached from tea leaves were PO_4_^−3^, SO_4_^−2^, F^−^, Cl^−^, and NO_3_^−^ [12]. Depending on the amount of the element that is leached during the preparation of the infusion, they are divided into three groups: highly-extractable (> 55%) – i.e., Cs, Rb, Na, Tl, K, Ni, Co; moderately-extractable (20–55%) – Mn, Mg, P, Zn, Al; and poorly-extractable (< 20%) – Cu, Mo, Y, Ba, Fe, Sr, Ca, Zr, Sn, lanthanides, Pb, Cd, Th, U, and W [13].

Tea leaves and infusions contain elements at the macro-, micro-, trace, and ultratrace levels. Spectroscopic methods of trace analysis enable their detection in broadly understood biological material. The most commonly used are atomic absorption spectrometry (AAS) with the flame (FAAS), electrothermal (ETAAS) and volatile hydrides generation (HGAAS) techniques, but also inductively coupled plasma spectrometry (ICP-AES), and inductively coupled plasma mass spectrometry (ICP-MS) [10, 11, 13]. The use of various statistical analysis methods enables and facilitates both the verification and interpretation of the obtained results.

Both tea leaves and their infusions contain elements necessary for the proper functioning of the body and participate in many life processes. But they also contain some dangerous elements such as Al [1, 9, 10, 13]. Tea is a popular and frequently consumed drink. Therefore, there is a need to estimate the amount of elements which may be intake with the beverage, as well as the impact of various conditions on their leaching and the mechanisms that accompany the process. Considering the need to maintain homeostasis in the body, not only the quantity but also the mutual quantitative proportions between the elements are important.

In our [14] previous work, we determined the content of elements – (Al, Ca, Cu, Fe, K, Mg, Mn, Na, and Zn) in green tea infusions and their interrelationships. However, in the present work, we attempted to determine the amount of the elements in black tea infusions brewed under the same conditions (time and pH). Quantitative analysis was made with the AAS method. Using various statistical analysis methods, we compared the infusions of both teas in terms of the number of leached elements depending on the brewing time and the pH value of the beverage as well their quantitative relations.

## Materials and Methods

### Instrumentation

An atomic absorption spectrometer AVANTA Ʃ (GBC Scientific Equipment Pty. Ltd, Australia) was used for trace analysis. The device is equipped with two burners (air- C_2_H_2_ and N_2_O-C_2_H_2_), graphite furnace GF3000, continuous flow hydride generator HG3000 with a cold mercury vapor concentrator MC3000, and ULTRA-PULSE background correction system. A flame atomizer was used in the instrumental analyses. The pH measurements were made using the pH-meter CP 501 (Elmetron, Poland). Tea samples were weighed on the analytical balance (ScalTec, Germany). Deionized water (0.06 µS/cm) needed for analyses was obtained from a deionizer produced by Cobrabid Aqua Company (Poland).

### Reagents

In analyses, the following reagents were used: stock solutions (Al, Ca, Cu, Fe, K, Mg, Mn, Na, Zn) (1.000 g/L, Fluka Chemie GmbH, Switzerland) for spectrometer calibration, 65% HNO_3_ Ultranal (Cheman Ciech, Poland), LaCl_3_ (Fisher Scientific, UK, 5000 µg/mL of La) and 5% CsCl (CPI International, USA) as buffers in Al, Ca, Mg, K, and Na in measurements.

## Procedures

### Sample Preparation

Black tea (Lipton Yellow Label) in the form of bags and leaves was purchased from a local store. Tea bags were weighed (1.997 ± 0.0201 g) and also samples of loose leaf (approximately 2 g as recommended by the manufacturer, i.e. 2.010 ± 0.014 g). Tea bags and leaves were poured with hot tap water (approximately 90 °C) in an amount of about 230 mL. Infusions were prepared in beakers within 5, 10 and 20 min. Next, they were transferred to volumetric flasks and filled up to the mark (250 mL) with tap water. At the same time, further infusions were prepared with the addition of an appropriate amount of HNO_3_ so that the pH value was close to 2, which approximately corresponds to the acidity of the infusion made with the addition of lemon. The infusions were made in 3 replicates (n = 36) and 4 tap water samples were treated as blanks. Next, the pH value was measured in infusions. The average pH of tea infusions from bags was 6.40 ± 0.08 and 2.17 ± 0.17, whereas for the loose tea—6.68 ± 0.17 and 2.20 ± 0.07. Successively, HNO_3_ was added in order to make the pH = 1.00 in all brew samples.

In the infusion samples prepared in this way, the content of elements was measured using the AAS method.

### Sample Analysis

The contents of elements were quantified by the flame technique (FAAS), under standard conditions. Parameters as well as instrumental analysis and validation methods have been described previously [14]. In summary, Ca, Cu, Fe, K (λ = 404.4 nm), Mg, Mn, Na (λ = 330.2 nm), and Zn amounts were measured in the air-acetylene flame, whereas Al (λ = 396.2 nm) concentration was determined in the N_2_O-acetylene flame LaCl_3_ (for Ca and Mg) and CsCl (for Al, K, and Na) buffers were used in the analytical procedures to minimalize chemical and ionization interferences. The accuracy of the measurements was verified based on the analysis of reference materials INCT-TL and SRM 1577b. The accuracy of the analytical procedures used was as follows: Al—117.9 ± 3.6%, Ca—103.8 ± 3.8%, Cu – 93.7 ± 2.2%, Fe – 98.1 ± 2.8%, K – 103.2 ± 0.9%, Mg – 95.2 ± 5.8, Mn – 97.5 ± 1.9%, Na – 94.2 ± 0.8% (SRM1575b), and Zn – 92,3 ± 1.0. Precision of analytical procedures was the following: Al – 1.6% Ca- 1.9%, Cu- 1.2%, Fe- 1.8%, K- 5.0%, Mg- 1.2%, Mn- 1.2%, Na- 2.8%, Zn- 1.0%.

### Statistical Analyses

Raw data were processed using Microsoft 365 (Excel) and the Statistica ver. 10 software package. The Shapiro–Wilk normality test was used for evaluating the distribution of variables. The Spearman test, for non-parametric data distributions, was applied to assess relationships between the pH value of the infusion and the content of extracted elements as well as their amounts in the infusions. The Kolmogorov–Smirnov test was employed to examine whether the content of elements in two groups differs significantly. The level of statistical significance was considered at p < 0.05.

Principal Components Analysis belongs to the statistical multivariate analytical methods. The PCA approach is based on the orthogonal, linear conversion of the input data set to new variables, which are not correlated and not always easy to interpret, commonly known as components or factors. This allows the PCA conversion to be presented graphically, and similarities and differences of input element concentrations reflecting their amount in the tested tea infusions or droughts can be easily seen.

## Results

### Element Content in Infusions

**Fig. 1:**
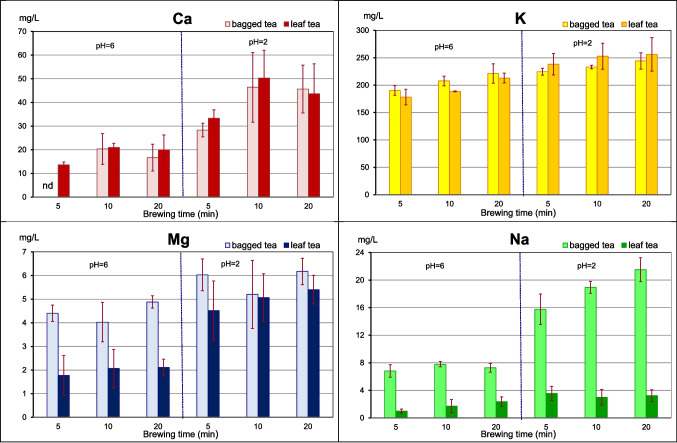
Concentration (mg/L) of macroelements in black teas infusions—without their content in tap water—($\overline{x }$ and sd)

**Fig. 2:**
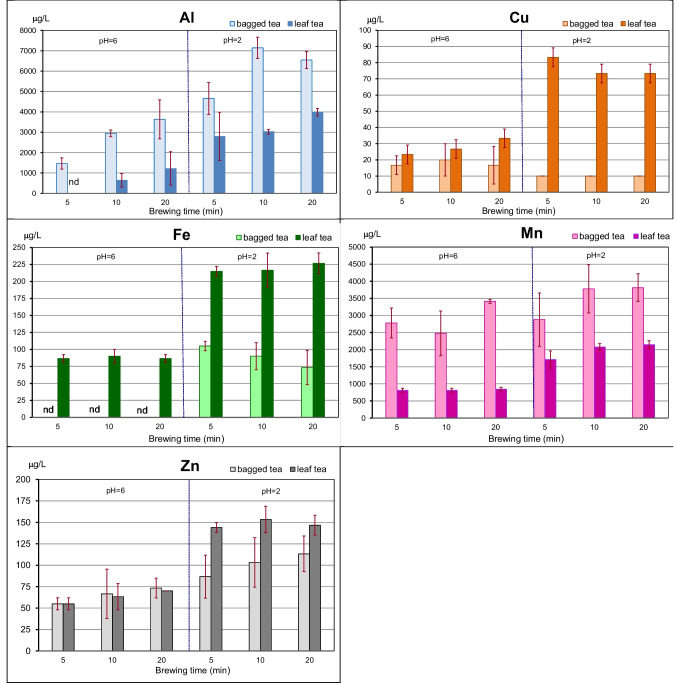
Concentration (µg/L) of trace elements in black teas infusions—without their content in tap water—($\overline{x }$ and sd)

The amounts of elements presented in Tables 1 and 2 as well as Figs. 1 and 2 are expressed as an arithmetic mean and standard deviation after subtraction their quantity in the tap water.

**Table 1:** Macroelement content released from 1 g of black tea into infusions after subtraction their quantity in the tap water, ($\overline{x }$ ± sd)

	pH	Ca mg	K mg	Mg mg	Na mg
B^a)^ 5	6	ND	23.6 ± 1.0	0.55 ± 0.04	0.85 ± 0.10
B 10	6	2.51 ± 0.80	25.7 ± 1.3	0.55 ± 0.09	0.97 ± 0.06
B 20	6	2.10 ± 0.62	27.5 ± 2.2	0.60 ± 0.03	0.90 ± 0.07
B 5	2	3.54 ± 0.34	28.3 ± 0.6	0.73 ± 0.08	1.98 ± 0.29
B 10	2	5.89 ± 1.80	29.4 ± 0.5	0.75 ± 0.16	2.39 ± 0.12
B 20	2	5.78 ± 1.28	30.8 ± 2.1	0.78 ± 0.07	2.77 ± 0.23
L^b)^ 5	6	1.70 ± 0.15	22.0 ± 1.9	0.22 ± 0.10	0.12 ± 0.03
L 10	6	2.61 ± 0.21	23.4 ± 0.1	0.26 ± 0.10	0.23 ± 0.17
L 20	6	2.50 ± 0.78	26.6 ± 1.1	0.26 ± 0.04	0.31 ± 0.08
L 5	2	4.13 ± 0.46	29.5 ± 2.3	0.56 ± 0.15	0.43 ± 0.12
L 10	2	6.28 ± 1.46	31.5 ± 2.9	0.63 ± 0.12	0.38 ± 0.14
L 20	2	5.41 ± 1.62	31.6 ± 4.1	0.67 ± 0.08	0.39 ± 0.12

^a)^bagged tea, ^b)^leaf tea

**Table 2:** Microelement content released from 1 g of black tea into infusions after subtraction their quantity in the tap water, ($\overline{x }$ ± sd)

	pH	Al µg	Cu µg	Fe µg	Mn µg	Zn µg
B^a)^ 5	6	222.4 ± 73.7	1.32 ± 0.13	ND	313.6 ± 8.8	6.80 ± 0.84
B 10	6	361.2 ± 14.6	1.24 ± 0.04	ND	306.8 ± 83.2	8.26 ± 3.50
B 20	6	410.3 ± 113.1	1.24 ± 0.01	ND	424.0 ± 6.7	9.10 ± 1.38
B 5	2	559.5 ± 83.0	1.25 ± 0.01	13.10 ± 1.04	360.6 ± 99.5	10.82 ± 3.22
B 10	2	900.9 ± 71.0	1.26 ± 0.01	12.68 ± 1.82	476.7 ± 91.8	13.07 ± 3.69
B 20	2	830.6 ± 54.3	1.26 ± 0.01	11.76 ± 1.25	482.7 ± 51.7	14.33 ± 2.61
L^b)^ 5	6	ND	2.90 ± 0.71	10.77 ± 0.68	101.2 ± 7.0	8.25 ± 0.63
L 10	6	123.3 ± 20.1	3.31 ± 0.73	11.18 ± 1.28	101.0 ± 6.9	7.87 ± 1.84
L 20	6	174.7 ± 70.7	4.16 ± 0.72	10.81 ± 0.72	105.6 ± 7.3	8.60 ± 0.17
L 5	2	311.2 ± 122.2	9.96 ± 0.01	26.75 ± 0.88	212.0 ± 29.2	21.15 ± 1.76
L 10	2	378.3 ± 13.9	9.15 ± 0.72	27.05 ± 3.13	259.7 ± 12.5	19.11 ± 1.88
L 20	2	552.2 ± 106.9	9.01 ± 0.56	27.97 ± 1.56	265.1 ± 10.12	18.10 ± 1.15

^a)^bagged tea, ^b)^leaf tea

In some infusion samples, the content of Al, Ca, and Fe was at the level of their amount in the tap water, so the differences between them were below the limit of detection (LOD), i.e. Al – 0.50 µg/mL, Ca – 0.02 µg/mL, and Fe—0.05 µg/mL. In such a situation, the amount of the element extracted from 1 g of tea was assumed as “not detected” (ND).

The average content of elements in 250 mL of tea infusion, together with tap water, was as follows (mg): Ca – 23.32 ± 4.05, Mg – 3.05 ± 0.32, K – 49.3 ± 9.6, and Na—4.64 ± 1.76, and (µg): Al – 1230 ± 560, Cu – 13.37 ± 6.74, Fe – 54.32 ± 26.50, Mn—581.6 ± 306.3, and Zn – 61.34 ± 6.74.

The highest amounts of K were determined in black tea infusions, while the lowest Cu quantities were found.

### Element Amount Leaching from 1 g of Tea

Expressing the amount of elements transferred to the infusion per 1 g of raw material facilitates comparative analysis between different tea samples as well as elements.

Among the elements analyzed, the largest amounts of K migrated into the solution (Tables 1 and 2), followed by Ca, Mg and Na. Next came Al and Mn, and then Fe and Zn. The least amount of Cu was found in the brews. The infusions prepared from tea bags contained more Al, Mg, Na, and Mn, approximately twice as much as in the infusions of loose leaf tea. Comparable amounts of K and Zn were leached from 1 g of both types of tea. 

The following Tables 3, 4 and 5 contain the results of the statistical analyses, taking into account their amount that was extracted per 1 g of tea.

**Table 3:** Analysis of the significance of differences between the groups depending on pH results, (Kolmogorov–Smirnov test)

	Al	Ca	Cu	Fe	K	Mg	Mn	Na	Zn
B, pH 2/6	0.01	0.005	0.1	-	0.01	0.005	0.1	0.001	0.1
L, pH 2/6	0.005	0.001	0.001	0.001	0.005	0.001	0.001	0.1	0.001
B vs L, pH = 6	0.01	0.1	0.001	-	0.1	0.001	0.001	0.001	0.1
B vs L, pH = 2	0.01	0.1	0.001	0.005	0.1	0.1	0.001	0.025	0.001

**Table 4:** Analysis of the significance of differences between the groups depending on time brewing results, (LSD test)

	Al	Ca	Cu	Fe	K	Mg	Mn	Na	Zn
B, pH = 6	1^*)^− 3^***)^	-	-	-	1–3	-	2–3	-	-
B, pH = 2	1–2^**)^, 3	-	-	-	1–3	1–3	-	1–3	-
L, pH = 6	-	-	-	-	1–3, 2–3	-	-	-	-
L, pH = 2	1–3, 2–3	-	-	-	-	-	1–2, 3	-	-

1^*)^− 5 min, 2^**)^- 10 min, 3^***)^- 20 min

**Table 5:** Results of correlation analysis between the amount of elements in infusions (Spearman rank correlation coefficients, r_s_)

	Al	Ca	Cu	Fe	K	Mg	Mn	Na	Zn
								pH = 6	
a) bags									
Al	1	0.88^*)^	− 0.54	-	0.52	0.02	0.10	0.52	0.06
Ca	0.89^*)^	1	− 0.59	-	0.39	0.12	− 0.20	0.71^*)^	− 0.07
Cu	0.51	0.48	1	-	− 0.44	0.06	− 0.22	− 0.28	− 0.33
Fe	− 0.60	− 0.48	− 0.30	1	-	-	-	-	-
K	0.32	0.18	0.07	0.60	1	0.36	− 0.71^*)^	0.00	0.61
Mg	0.43	0.51	− 0.02	− 0.65	− 0.07	1	0.54	0.12	0.87^*)^
Mn	0.86^*)^	0.76^*)^	0.40	− 0.80	0.36	0.74^*)^	1	− 0.45	0.71
Na	0.75	0.67	0.78^*)^	− 0.48	0.20	0.47	0.79^*)^	1	− 0.12
Zn	0.74	0.84^*)^	0.61	− 0.03	0.12	0.49	0.66	0.73^*)^	1
		pH = 2							
b) leaves									
Al	1	0.81^*)^	0.39	0.26	0.63	− 0.15	0.60	0.81^*)^	− 0.28
Ca	0.33	1	0.56	0.10	0.46	0.04	0.13	0.27	− 0.12
Cu	− 0.54	− 0.68	1	0.56	0.67^*)^	0.33	0.11	0.14	0.22
Fe	0.57	− 0.27	− 0.13	1	0.03	− 0.29	0.41	0.01	− 0.10
K	0.45	0.26	− 0.62	0.21	1	0.23	0.27	0.43	0.24
Mg	0.31	0.18	− 0.78^*)^	0.19	0.52	1	− 0.64	− 0.40	0.61
Mn	0.38	0.32	− 0.12	0.05	− 0.13	0.32	1	0.83^*)^	− 0.20
Na	− 0.70	− 0.10	− 0.46	− 0.70	− 0.15	0.20	− 0.20	1	− 0.37
Zn	− 0.47	− 0.82^*)^	0.96^*)^	− 0.25	− 0.61	− 0.69	− 0.18	− 0.30	1
		pH = 2							

^*)^ statistically significant value, p < 0.05

### Element content in Infusions According to their pH Value

The acidity of the tea infusion significantly affects the amount of extracted elements (Table 3, the Kolmogorov–Smirnov test). The acidity of the tea infusion significantly affects the amount of extracted elements. This especially applies to Al, Ca, K, Mg, and Na extracted from bags (Table 3). However, low pH significantly increased the amount of all elements, except Na, in the infusions prepared from leaf tea. At pH = 6, no significant differences in extraction between tea bags and loose-leaf tea were observed for Ca, K, and Zn, while at pH = 2, no significant differences were recorded for Ca, K, and Mg.

### The content of Elements in Infusions Depending on the Brewing Time

Tea brewing time significantly influenced the extraction of mainly Al, K and Mn and, to a lesser extent, Ca, Mg and Na (Table 4). In the case of Al, Ca and Mn, the differences were statistically significant between all-time groups: 5, 10, and 20 min, while K and Na was extracted the most after 20 min.

It appears, based on analysis of the differences in significance between the groups considering brewing time, that the time of 10 min is sufficient and often optimal for preparing tea infusions taking into account the degree of migration of such elements as Ca, Cu, Fe, K, Mg, Na, and Zn and, on the other hand, Al and Mn.

### Correlations Between the Quantity of Elements in Beverage

- Results of Spearman^’^s rank correlation analysis

Significant correlations occurred between Al and Ca in both bagged and loose tea infusions. Al in both types correlates moderately or strongly with most of the elements tested.

In infusions made from leaves, a smaller number of significant correlations were found between the elements compared to tea in tea bags. Pairs of correlating elements that appear in both bagged and loose leaf tea infusions are: Mg-Zn, Al-Ca, Zn-Cu, Al-K, and Al-Na (Table 5).

b) Results of PCA analysis

**Fig. 3:**
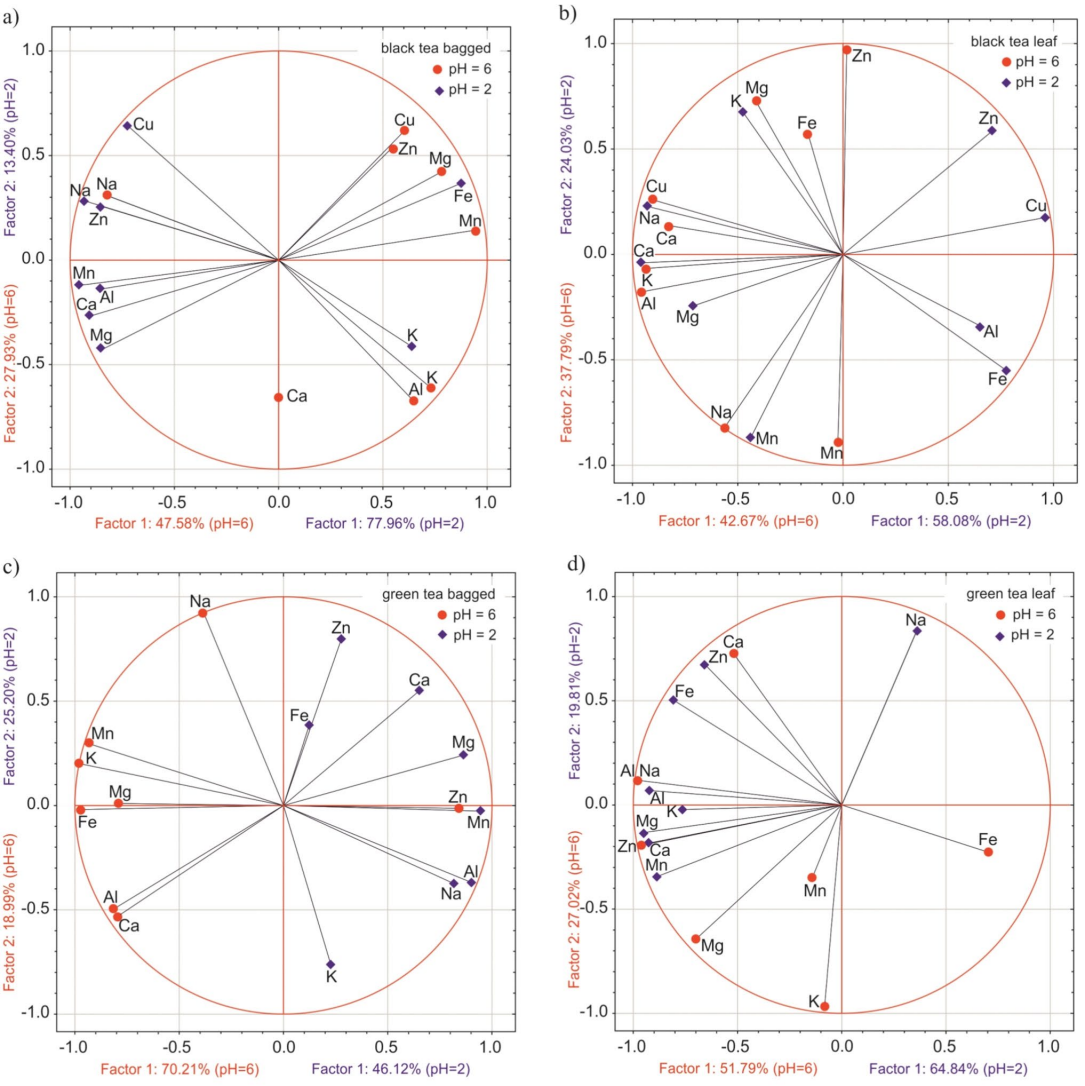
The influence of pH value on the interactions between elements extracted from 1 g of tea to 250 mL of infusion (PCA analysis)

Figure 3 illustrates the results of PCA analyses according to the influence of pH on the interactions between elements regarding their amounts extracted from 1 g of black tea in 250 mL beverage – bagged (a) and leaf tea (b) – as well as in green tea (c and d, respectively). The presented data are averaged over the brewing time.

In Fig. 3 (a and b), we can see the input variables – elements – located in the PCA unit circle for black tea infused for two tea acidities. The following rule applies to each PCA diagram: the closer the points representing input variables are, the greater the similarity and the stronger the positive correlation between them. If the vectors connecting the points (i.e., element content in the infusion) are rectangular, the variables are not correlated. But if the vectors lie along the same line but on the opposite sides of the PCA axis, the variables have a maximally negative correlation We can see that both for leaves and bags, pairs of strongly correlated elements (positively or negatively) appear for each tea pH. There are also non correlated elements in any case for tea acidity or tea input form. The same can be observed for green tea which is presented in Fig. 3 (c and d). In general, for black tea we found 24 element pairs positively correlated (correlation ≥ 0.8), 13 negatively correlated (≤ − 0.8), and 22 non-correlated element pairs (correlation ≤| 0.2|), while for green tea we have 19 element pairs positively correlated, only 3 negatively correlated, and 18 non-correlated element pairs (≤| 0.2|), respectively. If we look at tea pH values, we see more strong correlations for pH = 2 (37 positive and negative correlations) and less non-correlated pairs (16) as compared to black and green tea with pH = 6 (22 strong correlations and 24 non correlated element pairs).

### *Percentage of Elements Contained in 250 mL of Infusion in the Realization of the RDA *[18, 19]*.*

The amount of Mn in black tea infusion provides over 15% of this element (pH = 6) and over 22% in an acidic environment in relation to the daily requirement, just over 1% of K and also 0.5% and about 1% of Ca (Table 6). The remaining elements contribute from 0.06% (Na) to 0.83% (Cu) to the daily requirement. The significant quantitative advantage of K over Na is beneficial for health, in terms of preventing cardiovascular diseases.

**Table 6:** Percentage of elements contained in 250 mL of infusion in the realization of the RDA, (without their content in tap water)

	Ca	Cu	Fe	K	Mg	Mn	Na	Zn
pH = 6	0.46	0.35^a)^ 0.29^b)^	0.14^a)^ 0.20^b)^	1.42	0.27^a)^ 0.23^b)^	15.47	0.06	0.22^a)^ 0.17^b)^
pH = 2	1.04	0.83^a)^ 0.67^b)^	0.28^a)^ 0.40^b)^	1.72	0.43^a)^ 0.37^b)^	22.2	0.16	0.42^a)^ 0.33^b)^
Ref	19	19	19	19	19	19	18	19

^a)^women, ^b)^men

### Differences Between Element Content in Black and Green Tea Infusions

Black tea infusions contain higher amounts of Ca, Cu and Mn, and lower amounts of K, Fe, Mg and Na. The Zn content did not differ significantly (Table 7). Comparing infusions made from bags and leaves, we can state that black tea infusions prepared from bags contained more Al, Mg, Mn and Na, while loose leaf tea contained more Cu, Fe and Zn. Whereas green tea infusions prepared from bags contained more Al, Fe, Mn and Na, and leaf tea infusions—more K and Mg. Black tea infusions contain less K than green tea infusions. Perhaps this is due to the quality of the raw material from which the tea was produced, and the age of the plant, as well as the environmental conditions and the regions from which the tea originates.

**Table 7:** Analysis of the significance of differences in element contents extracted per 1 g between black and green teas, (Kolmogorov-Smirnov test, (p < 0.05)

	Al	Ca	Fe	K	Mg	Mn	Na	Zn
B, pH = 6	> 0.1	0.025	-	0.001	0.001	0.025	0.001	> 0.1
B, pH = 2	0.005	> 0.1	0.005	0.001	0.001	0.025	0.005	> 0.1
L, pH = 6	> 0.1	0.001	0.01	0.001	0.001	0.001	0.025	0.025
L, pH = 2	> 0.1	> 0.1	0.005	0.001	0.001	0.001	> 0.1	0.001

## Discussion

We should pay special attention to the amount of two elements: Al and Mn. In black tea infusions, similarly to green tea, large amounts of Al were found, especially in acidic infusions. Comparable amounts, although in some cases slightly smaller ones, were found for Mn. Both metals are known primarily as neurotoxic elements [15–17]. Their harmful effects on living organisms have been and are the subject of much research and many publications. However, Mn deficiency is also harmful because it is a cofactor for many important enzymes, including Mn superoxide dismutase, arginase, and pyruvate carboxylase. It is also required for ensuring properly functioning processes in the skeletal and immune system [16, 17]. A Tolerable Upper Intake Level for Mn is estimated at 11 mg/day for adults and 2–3 mg/day for children [18], whereas, according to the EFSA [19] recommendations, Adequate Intake for the element is established at 0.5–1.0 mg/day for children and 3.0 mg/day for adults. As far as Al is concerned, the Tolerable Weekly Intake (TWI) for the element should not exceed 1 mg/kg bw/week [15]. 1 L of bagged tea in an acidic environment can provide almost 4 mg of Mn (Fig. 2) and 7 mg of Al (Fig. 2).

The oral absorption of Al and Mn is rather small, but excessive exposure (e.g., due to drugs and certain environmental/professional conditions) to both elements is dangerous, especially for children and people with absorption and excretion disorders.

Generally, the results obtained in this study regarding the content of Al and Mn in black tea infusions are similar to those presented by other authors [9, 20–24]. According to Pękal et al. [25] about 10–19% of total Al content in brews occurs as cationic species and 28–33% as hydrolysable polyphenolic compounds. Whereas, Mn is present in cationic form in 58–71% of its total amounts in infusion, while also bound with the polyphenolic ligands. Both metals belong to the group of elements with a high extraction efficiency, and is established for Al—36 ± 2%, and Mn – 29% ± 7 [10].

The amounts of toxic and health-hazardous elements, e.g., Pb, Cd, As, in tea infusions are very small or sometimes below the LOD [10, 13, 20, 22], although authors of some studies (including our own unpublished data) have determined small amounts of Ni (0.026–0.269 mg/L) and Cr (0.04–0.42 mg/L) [20].

Other studied elements play key roles in the human body. Generally, they are components of bone tissue (Ca, Mg), are part of biologically active compounds (e.g. enzymes, hormones, transporting proteins, neurotransmitters, hemoglobin (Ca, Cu, Mn, Fe, Mg, Zn), participate in water and electrolyte management, maintain acid–base and osmotic balance (K, Na, Ca, Mg), and of various importance (e.g. redox reactions, energy and oxygen transfer, nutrient metabolism (Cu, Fe, Mg, Mn, Zn) [18, 19].

Research results regarding the content of elements in black tea infusions under the conditions of this experiment are similar to the data presented by other researchers [9–11, 13, 21, 26], (Table 8).

**Table 8:** Content of elements in black tea infusions, (µg/L)

Ca	K	Mg	Na	Al	Cu	Fe	Mn	Zn	Ref
57,130	306,350	18,730		5620					9
2450 ± 550		9400 ± 200		3200 ± 200	76 ± 17	41 ± 5	2200 ± 500	140 ± 30	10
				1800 ± 100	71 ± 2	46 ± 4	2650 ± 50	170 ± 9	11
4030 ± 20	305,000 ± 21,000	19,600 ± 600	717 ± 70	5540 ± 240	102 ± 4	180 ± 30	5160 ± 180	288 ± 2	13
5460 ± 3420		6960 ± 1000		4610 ± 780	67.4 ± 1.1	19.4 ± 4.6	1710 ± 580	87.2 ± 5.7	21
10,000		15,000	1000	6000	10	60	3000	200	26

Regarding the observations of other authors, factors such as environmental conditions, treatments used during tea cultivation and production, species and the age of the plant largely determine the amount of elements in the infusion [1, 20, 27–29].

We have noticed, like some authors [22, 28], that in the case of both black and green tea, there are differences in the content of elements in loose-leaf and tea bag infusions. Like us, these authors determined higher amounts of Al, Mn, Ca, Mg but also K and Fe in Lipton tea infusions prepared from the bags. Differences are related, among others, to the worse type of raw material used to produce tea bags, but also the possibility of the presence of contaminations.

The amount of substance (element) diffusing into the solvent (boiling water) is determined by the difference in its concentration in both phases, the contact surface of the phases, the speed of phase movement, the extraction time, and temperature. Another important factor is the solubility of compounds in water, which influences the leaching process. Taşcioğlu and Kök [30] found significant differences in the content of Cu, Fe, Ni and Cr in black and green tea infusions at temperatures of 18^0^C, 40^0^C, 60^0^C and 80^0^C and different strengths of the infusions (1% and 8%). Quantitative changes in the content of elements depending on the tea-to-water ratio were also described by other authors [31]. In the case of Al and Ca, the influence of the chemical composition of water (the degree of mineralization) on their migration into infusions was described. They stated that higher mineralization reduces the amount of extracted Al and organic matter, and some amount of Ca may be bound to cell membrane pectins. Unfortunately, there is little information from research conducted on the influence of temperature and chemical composition of water on the amount of elements leached into the tea infusion.

We know a bit more about how time and water pH values determine these amounts. Regarding the work of Saletnik et al. [9] adding lemon juice increases the amount of K, Mg, P and Al, and especially Ca – even as much as approximately three times in the tea infusion. The amount of Al increased by as much as 1.5 times. The Mg increase was at the level of 5.53 to 9.91 mg/L. Street et al. [32] reported that in some cases the addition of lemon juice may affect Al speciation in tea beverages. Similar observations were made by other authors [33], i.e., the addition of citric acid changes the distribution of Al in infusions, increasing the amount of free ions of the element, as well as the amount of Cd, Pb and Mn.

Street et al. [32] measured generally larger amounts of Al in infusions of both black and green tea after 5 min (1.14–4.91 mg/L and 0.284–7.83 mg/L, respectively) and 60 min of brewing time (1.81 mg/L and 15.0 mg/L, respectively). However, Saletnik et al. [9] noted higher amounts of Ca, K, Mg, P and Al (generally 15–20%) after 15 min of brewing compared to 5 min of extraction. Atasoy et al. [34] examined the influence of time (2.5, 10, 30, 45 and 60 min) and tea concentration (1%, 2% and 3%) on the amount of leached elements (Al, Cd, Cr, Cu, Fe, Hg, Mn, Ni, Pb and Zn) for the infusion. In general, they stated that longer time brewing times and high tea concentrations result in higher metal content in beverages. Özcan et al. [35] determined that a brewing time of 10 min is sufficient to leach most of the 27 tested elements.

The method of preparing the infusion, the weight of the tea from which the infusion was made, the volume of water, the degree of its mineralization and temperature, brewing time, the pH value of solvents determine the amount of migrating elements. This is why there are differences in the data found in the literature.

Elements participate in many life processes. Their mutual relations, including quantitative ones, ensure the proper occurrence of biochemical reactions. The mutual relations may be synergistic or antagonistic. Therefore, their proper quantitative proportions in our diet are important. Several authors have studied correlations between elements in their research. Ashraf and Mian [36] stated strong correlations between Fe–Cr, Fe-Cd, and Pb-Cu pairs in black tea samples. Statistically significant correlations between the contents in the raw material and in the infusions of black tea (bagged and loose leaf) were determined for Na, P, and F by Klink et al. [28]. Strong correlations between the content of N, P, K, Ca, Mg and Zn in tea leaves and the amount of polyphenols were determined by Tseng and Lai [37].

Though tea infusions provide rather small amounts of nutritional elements to the daily diet, they are a valuable supplement. Their advantage is also found in their significant K content compared to Na, taking into account their role in the prevention of cardiovascular diseases. However, the intake of Al and Mn in tea infusions should be controlled.

Green and black teas differ in their chemical composition, especially in the content of catechins and complex polyphenols [1, 3, 4, 6]. Differences between teas also concern the elemental composition of tea leaves, mostly elements such as Al, Mn, and Ca. The observed variations by the authors may result from environmental conditions, technological processes, method of making the infusion and the age of the leaves [2–4, 13, 29, 38]. While, parameters of tea brewing on chemical composition, sensory evaluation and health efficacy have been described in detail by Liu et al. [39]. Some authors [2, 4, 9] report existing variations in the content of elements in green and black teas, while others [29] did not determine any significant differences. However, we can observe certain trends regarding the quantitative elemental composition of tea infusions, in both green and black tea infusions.

## Conclusions

The content of elements in black tea infusions (bagged and leaf) was examined depending on the brewing time and pH value. The amount of extracted elements was in the following sequence: K, Ca, Mg, Na, Al, Mn, Zn, Fe, and Cu. This order is in accordance with data presented in the literature. Tea infusions are a valuable but rather complementary source of elements in the diet. However, the possible amount of Al and Mn consumed with this drink should be taken into account. The quantitative proportion between K and Na is good for the prevention of cardiovascular diseases. Both brewing time and pH influenced the metal content in the infusions. Generally, a longer brewing time and pH = 2 (compared to pH = 6) increased their quantity. Taking into account the possibility of migration of elements into the infusion, the time of 10 min seems to be optimal, also due to the possibility of limiting Al leaching. Under the conditions of the study, differences were also observed in the amount of elements migrating to infusions from bagged and leaf tea. It has been found in infusions that some elements correlate with each other, for example: Al-Ca, Al-Mn, Mg-Zn, Ca-Mn, Ca-Zn. However, in our research, statistically significant differences in the amount of leached elements in black and green tea infusions were shown, among other, more Al and Mn migrate from green tea, both from leaves and tea bags. Moreover, in both cases, a relevant influence of the brewing time and pH value on the mineral composition of the infusions was found. The data presented by authors who tested the content of elements in infusions prepared from different types of tea and under different conditions are similar. To this end, we compared certain parameters (e.g., bagged vs. loose leaf tea) and conditions (e.g., brewing times, pH value) that influence the level and type of elements extracted from black tea infusions. This enables the nutritional value of tea to be assessed as it is frequently consumed around the world. Research on this topic is important and necessary as it allows us to determine the optimal conditions for preparing infusions of various types of teas.

In summary, the influence of several factors determining the amount of elements migrating from black and green tea to the infusion was comprehensively determined. This allowed us to assess the nutritional value of the infusions in terms of the amount of elements, but also to assess the risk resulting from the presence of those hazardous to health. This is particularly important, considering the fact that tea is a widely consumed beverage in the world.

## Data Availability

No datasets were generated or analysed during the current study.
